# Simultaneous Bi‐Jaw Reconstruction: Double Free Fibula Flap Following Resection of Synchronous Oral Malignancy

**DOI:** 10.1002/hed.70248

**Published:** 2026-03-26

**Authors:** Subhendu Khan, Varun Saini, Vikram Singh, Ashish Gulia

**Affiliations:** ^1^ Department of Plastic and Reconstructive Surgery Homi Bhabha Cancer Hospital and Research Centre (Unit of Tata Memorial Centre, Mumbai) New Chandigarh Punjab India; ^2^ Department of Surgical Oncology Homi Bhabha Cancer Hospital and Research Centre (Unit of Tata Memorial Centre, Mumbai) New Chandigarh Punjab India

**Keywords:** bi‐jaw reconstruction, double free fibula flap, mandibulectomy and maxillectomy, microvascular reconstruction, synchronus oral cancer

## Abstract

**Background:**

Simultaneous reconstruction of extensive maxillary and mandibular defects in oncological cases is a complex surgical challenge.

**Methods:**

We report a case of a 56‐year‐old female with synchronous oral malignancy involving upper and lower alveolus. Simultaneous double free fibula osteo‐cutaneous flaps were used for reconstruction of upper and lower alveolus reconstruction.

**Result:**

We were able to circumvent this challenging case with the use of double fibular free flaps. The reconstruction successfully restored form, function, and aesthetics.

**Conclusion:**

This case describes the first reported use of double fibular flap for simultaneous upper and lower jaw reconstruction in a case of synchronous oral malignancy.

## 1 Introduction

Simultaneous reconstruction of extensive maxillary and mandibular defects following oncological resection is a complex and rare surgical challenge. It requires meticulous planning and execution. Traditional approaches have evolved from staged reconstruction to the use of vascularized bone flaps combined with non‐vascularized bone graft. However, the long‐term viability of non‐vascularized grafts is limited, particularly in the context of postoperative radiation therapy. The fibula is the most commonly used bone for osseous reconstruction due to its length, robust bone quality, and adaptability for multiple osteotomies, as well as its ability to support dental implants [1]. While the use of double free flaps has been well‐documented for addressing extensive composite mandibular defects [2], the simultaneous use of bilateral fibular flaps for maxillomandibular reconstruction in oncological cases remains exceedingly rare. Nisanci et al. [3] reported the use of three simultaneous free flaps, including bilateral fibular and radial forearm flaps, for middle and lower third of facial reconstruction following traumatic injury. However, the application of bilateral fibular flaps specifically for simultaneous maxillary and mandibular defects in cases of synchronous lesions in cancer has not been widely reported.

We describe the simultaneous transfer of bilateral fibular flaps as double free flaps for the reconstruction of maxillomandibular defects following the resection of synchronous malignant lesions involving both upper and lower alveolus. This approach allowed for comprehensive reconstruction of the maxilla and mandible in a single‐stage operation, providing a novel solution for managing extensive composite defects in oncological patients.

## 2 Case

**FIGURE 1:**
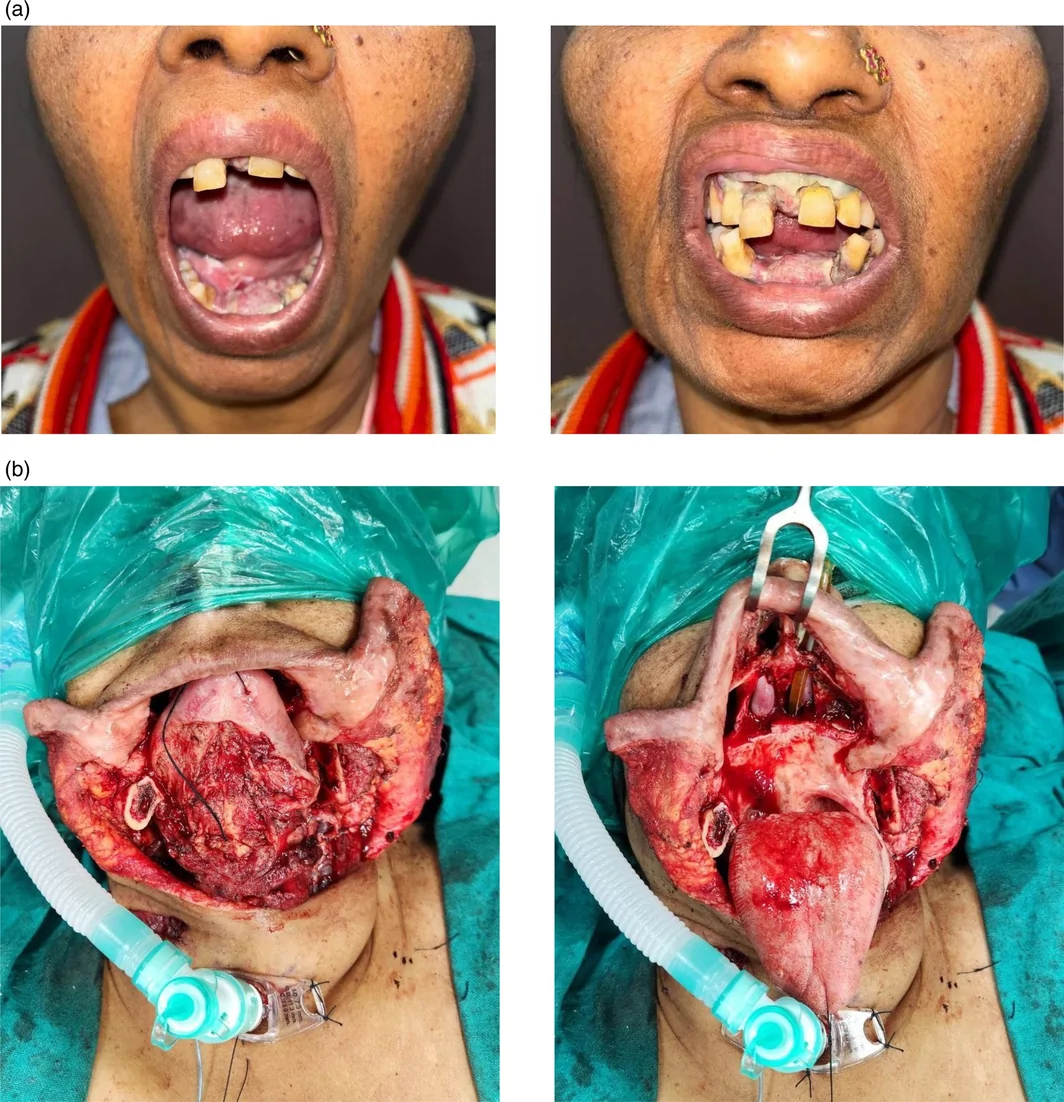
(a) 56‐year‐old woman with malignant lesions involving both the upper and lower alveolus. (b) Intra‐operative picture following resection of upper and lower alveolus.

A 56‐year‐old woman from North India presented with suspected malignant lesions involving both the upper and lower alveoli (Figure 1). The diagnosis was confirmed through biopsy and radiological imaging. The tumor was found to involve the upper maxillary alveolar ridge, affecting the bilateral central and lateral incisors and the lower alveolus, involving the central segment of the mandible. The surgical plan included segmental mandibulectomy and upper alveolectomy, followed by reconstruction using bilateral fibular flaps. After tumor resection, the defect spanned the central arches of both upper and lower alveolus (Figure 1b). The mandibulectomy defect extended short of the angles on both sides and included significant involvement of the floor of the mouth. The maxillary defect affected the upper central alveolus and the anterior part of the hard palate.

## 3 Surgical Technique

### 3.1 Mandibular Reconstruction

The left fibular osteo‐cutaneous flap was used to reconstruct the mandible, with anastomosis performed in the right neck to allow the skin paddle to naturally fall into the floor of the mouth. The reconstructed mandible measured 3.5 cm for the right body, 2.5 cm for the central segment, and 5.5 cm for the left body. A skin paddle of 10 × 6 cm was used to reconstruct the floor of the mouth. Two closing wedge osteotomies were performed, and fixation was achieved using titanium miniplates and screws. Anastomosis of the peroneal artery and vein was completed on the right side of neck. The peroneal artery was anastomosed with facial artery and the peroneal vein was anastomosed with a tributary of the internal jugular vein.

### 3.2 Maxillary Reconstruction

**FIGURE 2:**
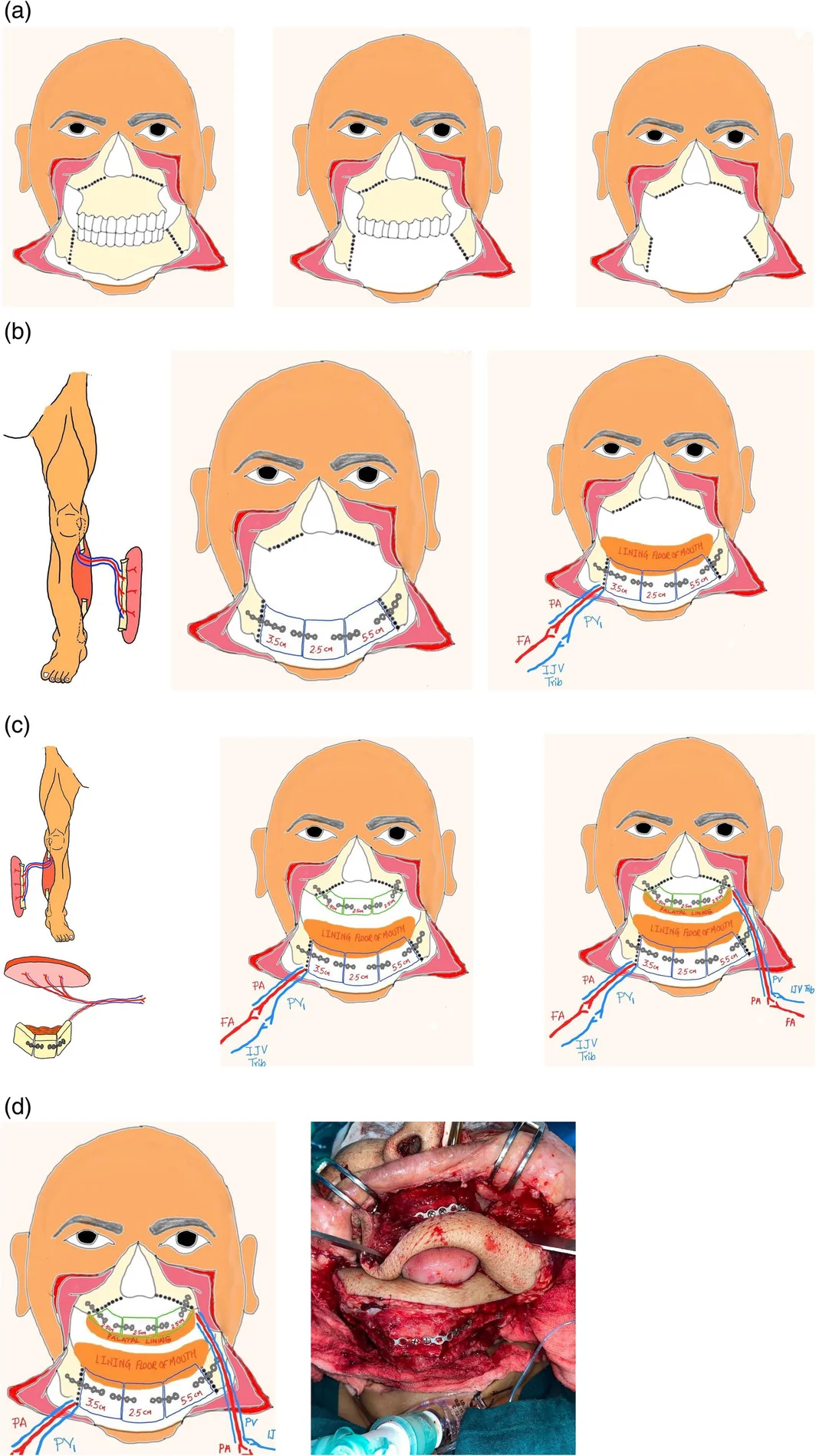
(a–d) Diagrammatic schematic flow of steps of reconstruction. (a) (left) Diagram showing preoperative tentative cut margin in the upper and lower alveolus. (Middle) Defect following resection of lower alveolus (right). Defect following resection of upper alveolus. (b) For lower alveolus reconstruction harvested fibula from the left leg was reconstructed as neo‐mandible following plate fixation (middle), following fixation partial inset in the floor of mouth was done and anastomosis done in the right neck (right). (c) The right fibular osteo‐cutaneous flap was used for reconstructing the upper alveolus. A 6 × 5 cm skin paddle was designed based on a prominent perforator and separated from the bone to provide flexibility for inset. Reconstructed frame measured 2.5 cm each for the right body, central segment and left body. Following plate fixation inset done in palate and anastomosis done in the left neck. (d) Post fixation diagram showing lie of the pedicle and skin paddle. Intra operative picture following fixation of both fibula and prior completion of inset.

The right fibular osteo‐cutaneous flap was used for reconstructing the upper alveolus. The reconstructed frame measured 2.5 cm each for the right body, central segment and left body. A 6 × 5 cm skin paddle was designed based on a prominent perforator and separated from the bone to provide flexibility for inset. The skin paddle was secured to the nasal floor to separate the nasal and oral cavities, preventing infection. It was partially de‐epithelialized for lip inset and used for recreating the palatal lining. Fixation was achieved using titanium miniplates and screws. The flap pedicle was tunneled to the left side of neck for anastomosis. The peroneal artery was anastomosed with left facial artery and the peroneal vein was anastomosed with the tributary of the internal jugular vein. A schematic flow of steps of reconstruction is shown in Figure 2.

**FIGURE 3:**
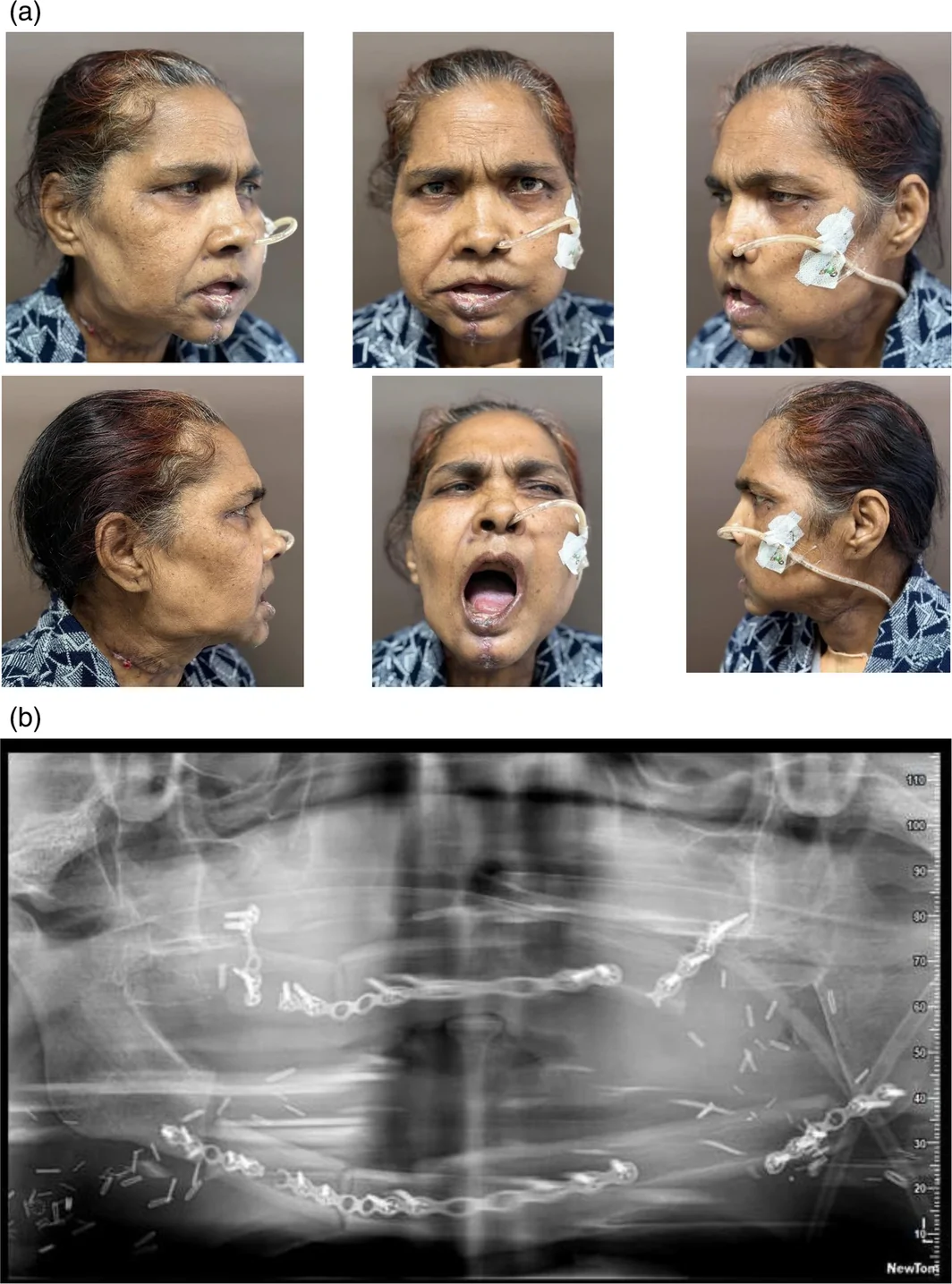
(a) Postoperative picture of the patient on follow up. (b) Postoperative orthopantomogram (OPG) of the patient.

The entire reconstruction procedure was completed within 8 h as a single‐stage surgery. There were no systemic or microvascular complications in the postoperative period. The patient was successfully discharged from the hospital after 2‐week postsurgery. The patient followed a strict rehabilitation protocol with a dedicated physiotherapy team, beginning partial weight‐bearing 2 weeks after surgery and progressing to full weight‐bearing by the fourth week. Both the donor and recipient sites healed satisfactorily. Radiation therapy was initiated according to the institutional protocol. An orthopantomogram (OPG) was obtained as baseline record. (Figure 3b).

## 4 Discussion

Simultaneous reconstruction of extensive maxillary and mandibular defects presents a significant challenge in head and neck reconstructive surgery. The use of double fibular free flaps in this case demonstrates a viable and innovative approach for achieving both functional and aesthetic reconstruction, providing robust structural support and favorable contour restoration. This approach is particularly pertinent in cases where a single flap would fail to meet the complex requirements of length, mobility, and dual‐plane reconstructions.

The fibular flap has long been a preferred choice for mandibular reconstruction due to its reliability and versatility. Sadove and Powell [4] first described the use of a single free fibular flap for simultaneous maxillary and mandibular reconstruction. In cases with extensive defects, the use of multiple simultaneous free flaps has become an essential technique in head and neck reconstruction. These are typically combinations of bone flaps and soft tissue flaps. Tel et al. [5] described the application of double osseous flaps, specifically a fibular flap and a deep circumflex based iliac artery‐based osseous flap, for simultaneous midfacial and mandibular reconstruction. Nisanci et al. [3] reported a case in which three simultaneous free flaps, including bilateral fibular flaps and a radial forearm flap, were utilized for middle and lower third facial reconstruction following a gunshot injury. Burt et al. [6] described the reconstruction of facial deformities using multiple soft tissue flaps in a pediatric case of facial trauma caused by a dog bite. However, the simultaneous use of bilateral fibular flaps for oncological reconstruction, as presented in this report, remains a rare occurrence in the literature.

The fibula remains the gold standard for mandibular reconstruction due to its robust cortical structure, predictable vascular anatomy, and ability to tolerate multiple osteotomies and support dental implants [1]. Sadove and Powell were among the first to describe the simultaneous use of a single free fibular flap to reconstruct both the maxilla and mandible by segmenting the fibula. While effective in some scenarios, the technique is limited by the length of the intervening pedicle required to position the segments, as well as the reduced bone stock available for each site. In this case, bilateral central alveolar defects involving both the upper and lower alveolus required extensive reconstruction. A total of 21 cm of vascularized bone was necessary, along with independent pedicles to enable precise three‐dimensional positioning and sufficient pedicle length to reach the neck for reliable microvascular anastomosis. Additionally, ample soft tissue was needed to ensure dependable mucosal lining reconstruction. These combined demands exceeded the capacity of a single fibular free flap, thereby necessitating the use of a dual‐flap approach.

Double fibular free flaps offer several critical advantages over alternative approaches. Unlike the segmented single fibula technique described by Laure et al. [7] and Punpale et al. [8], which can result in limited freedom of flap positioning, the use of two independent fibula flaps allows for precise reconstruction in two separate planes. This eliminates constraints related to pedicle length and mobility, ensuring better alignment and integration of the reconstructed segments. Additionally, simultaneous reconstruction with bilateral fibulas obviates the need for sequential or staged procedures, reducing the overall treatment timeline and potentially improving patient outcomes.

Alternative strategies, such as the use of combined flaps—including the deep circumflex iliac artery flap or scapular osseous flaps—have been described for complex reconstructions. For instance, Tel et al. [5] reported the use of double osseous flaps for midface and mandibular reconstructions, while Nisanci et al. [3] demonstrated reconstruction of extensive defects following trauma with three simultaneous free flaps, including bilateral fibular flap (flow‐through) and radial forearm flaps. While effective, these techniques are associated with increased operating time, donor site morbidity, and technical complexity. The scapular flap, though capable of providing to independent bone segments, is less suited to tolerate multiple osteotomies and requires patient repositioning. If the pedicle falls short, it requires vein graft, which further complicates an already lengthy procedure. Mericli et al. [9] described simultaneous vascularized bony reconstruction of the maxilla and mandible using two free bone flaps from a single fibula, which were vascularized via vein grafts, and an anterolateral thigh (ALT) flap was used for external cheek defect.

In the present case, the decision to use bilateral fibular flaps addressed several critical challenges. The neo‐mandible was reconstructed with left fibula, and the pedicle was oriented to facilitate tension‐free intraoral lining inset [10]. The right fibula was used for maxillary reconstruction, with its skin paddle modified for palatal lining to separate the nasal and oral cavities and prevent leakage during swallowing. The independent use of bilateral fibular flaps allowed for precise alignment and adequate soft tissue coverage, critical for functional restoration. Microvascular anastomosis was kept simple by choosing two independent units with anastomosis in the bilateral neck. By avoiding the use of non‐vascularized bone grafts, which are prone to failure when postoperative radiation is required or in irritated tissues, this approach ensured long‐term viability and supported future osseointegrated dental implants, as highlighted by Foster [11] and Pogrel et al. [12].

The primary limitations of this technique include increased operative time, additional donor‐site morbidity, and the loss of the fibula as a future reconstructive option. Although 3D‐printed, patient‐specific models may enhance preoperative planning and reduce operative duration, this technology was not available at our institution at the time and therefore could not be incorporated into the treatment plan. These challenges were mitigated by employing a two‐team approach, allowing simultaneous flap harvesting and reconstruction and thereby reducing operative time. Additionally, the use of venous coupler devices improved efficiency during microvascular anastomosis. Regular physiotherapy follow‐up ensured early recovery of full mobility.

To our knowledge, this report represents one of the few documented cases of simultaneous bilateral fibular free flap reconstruction for maxillomandibular defects. The successful outcome demonstrates the viability of this approach in addressing complex defects, particularly in cases where the other techniques fall short. The establishment of a robust bony framework with adequate soft tissue coverage not only simplifies future minor revisions but also enhances the patient's quality of life by enabling early oral feeding and facilitating dental rehabilitation.

## 5 Conclusion

Simultaneous double fibular free flap reconstruction offers a reliable and effective solution for extensive maxillomandibular defects, providing superior functional and aesthetic outcomes. With advancements in microsurgical techniques and technology, such complex reconstructions can be performed with increasing precision and predictability, setting a new standard for managing challenging head and neck defects.

## Author Contributions

Concept, design, analysis and interpretation of data, draft manuscript and final approval of manuscript, and reconstructive surgeon: Varun Saini and Subhendu Khan. Onco‐surgical resection plan: Vikram Singh and Ashish Gulia.

## Funding

The authors have nothing to report.

## Ethics Statement

This work is entirely original and was exempt from institutional IRB approval. No animals were involved in this study.

## Consent

Complete written informed consent was obtained from the patient for the publication of this case report and accompanying images.

## Conflicts of Interest

The authors declare no conflicts of interest.

## Data Availability

The data that support the findings of this study are available from the corresponding author upon reasonable request.
